# Declaration: Novel SLC3A1 mutation in a cystinuria patient with xanthine stones: a case report

**DOI:** 10.1186/s12894-023-01300-y

**Published:** 2023-07-31

**Authors:** Peide Bai, WenZhao Zhang, Longhui Lai, Haichao Huang, Jiaxuan Qin, Bo Duan, Huiqiang Wang, Yuedong Chen, Yuanyuan Jia, Jinchun Xing, Tao Wang, Bin Chen

**Affiliations:** 1 Department of Urology, The First Affiliated Hospital of Xiamen University, Xiamen, China; 2 Department of Pediatric Surgery, Women and Children’s Hospital, School of Medicine, Xiamen University, Xiamen, China; 3 GloriousMed Clinical Laboratory (Shanghai) Co., Ltd, Shanghai, China

**Keywords:** *SLC3A1*, Cystinuria, Xanthine stones, Genetic testing

## Abstract

**Background:**

Cystinuria and xanthinuria are both rare genetic diseases involving urinary calculi. However, cases combining these two disorders have not yet been reported.

**Case Presentation:**

In this study, we report a case of cystinuria with xanthine stones and hyperuricemia. The 23-year-old male patient was diagnosed with kidney and ureteral stones, solitary functioning kidney and hyperuricemia after admission to the hospital. The stones were removed by surgery and found to be composed of xanthine.

**Conclusion:**

Genetic testing by next-generation sequencing technology showed that the patient carried the homozygous nonsense mutation c.1113 C> A (p.Tyr371*) in the *SLC3A1* gene, which was judged to be a functionally pathogenic variant. Sanger sequencing revealed that the patient’s parents carried this heterozygous mutation, which is a pathogenic variant that can cause cystinuria. The 24-h urine metabolism analysis showed that the cystine content was 644 mg (<320 mg/24 h), indicating that the patient had cystinuria, consistent with the genetic test results. This case shows that cystinuria and xanthine stones can occur simultaneously, and provides evidence of a possible connection between the two conditions. Furthermore, our findings demonstrate the potential value of genetic testing using next-generation sequencing to effectively assist in the clinical diagnosis and treatment of patients with urinary calculi.

## Background

Cystinuria refers to the disorder of cystine reabsorption by renal tubular epithelial cells at the proximal end of the kidney and small intestinal mucosal epithelial cells. This condition results in an increase in the concentration of cystine in the urine, and the formation of cystine stones due to its low solubility. All patients with cystine stones have a high risk of recurrence; therefore, this disorder has become a focus of clinical research. Cystinuria is a rare autosomal recessive or incompletely dominant inherited disorder [1]. Studies have shown that the disease is caused by mutations in the *SLC3A1* gene (located on chromosome 2p21) encoding neutral and basic amino acid transport protein rBAT and the *SLC7A9* gene (located on chromosome 19q13) encoding b(0,+)-type amino acid transporter 1 [2, 3]. The discovery of these two genes changed the traditional classification of cystinuria (type I, type II and type III). According to the mutation types of these two genes, Dello Strologo reclassified cystinuria into type A (two mutations on *SLC3A1*), type B (two mutations on *SLC7A9*), and type AB (one mutation on each of *SLC3A1* and *SLC7A9*) [4, 5]. This classification also provides a theoretical basis for genetic testing to assist clinical diagnosis.

Xanthinuria is a urinary system disorder caused by abnormal purine metabolism and excessive xanthine in the urine. Hereditary xanthinuria is divided into three types. Type I is a deficiency of xanthine dehydrogenase/xanthine oxidase (XDH/XO) caused by mutations in the *XDH* gene, although aldehyde oxidase (AOX) activity is normal. Type II is the result of a double deficiency in XDH/XO and AOX caused by mutations in the molybdenum cofactor sulfurylase (*MOCOS*) and *AOX1* genes. Type III is due to defects in the synthesis of molybdenum cofactors, leading to the lack of molybdenum cofactors associated with sulfite oxidase (SO), XDH/XO and AOX activities. The genes encoding molybdenum cofactor biosynthesis enzymes are *MOCS1*, *MOCS2* and *GPHN* [5–7]. The lack of these enzymes leads to a decrease in serum/urine uric acid levels and an increase in xanthine levels, resulting in xanthinuria.

Both cystinuria and xanthinuria are relatively rare diseases, and no cases of cystinuria with xanthine stones have been found clinically. In this study, genetic testing by next-generation sequencing (NGS) revealed that a patient with xanthine stones carried a genetic mutation indicative of cystinuria, which was confirmed by 24-h urine metabolism analysis. According to the 24 h urine metabolism analysis, the 24 h urine output is 2200 ml, oxalic acid 16 mg/24h, citric acid 387 mg/24h, cystine 644 mg/24h, urine chloride 189.2 mmol/24h, urine potassium 42.3 mmol/24h, urine sodium 182.6 mmol/24h, urinary calcium 0.66 mmol/24h, urinary phosphorus 8.36 mmol/24h, urinary magnesium 2.42 mmol/24h, urinary creatinine 12.10 mmol/24h, urinary uric acid 1.87 mmol/24h. In addition, routine biochemical findings suggest that blood calcium is 2.54 mmol/L and blood uric acid is 508.9 umol/L. Here, we report the follow-up results to provide references for exploring the causes of cystinuria and xanthinuria and the potential of genetic testing techniques for assisting the clinical diagnosis of urinary calculi.

## Case presentation

The 23-year-old male patient was admitted to the First Affiliated Hospital of Xiamen University on April 26, 2020 with the main complaint of left back pain for two days. Computed tomography of the abdomen showed multiple stones in the left kidney. The patient had a medical history of laparotomy and urinary stone removal in another hospital 10 years previously. On December 10, 2018 and September 16, 2019, left kidney and ureteral calculi lithotripsy on the left side were performed using a flexible ureteroscope in our hospital. Routine analysis of stones by infrared spectroscopy analysis system showed that the stones were composed of xanthine. After completing the relevant examinations on his most recent admission, the patient was diagnosed with a solitary kidney and ureteral stone incarcerated by hydronephrosis. On April 27, 2020, the patient underwent left transurethral ureteroscope laser lithotripsy, followed by left side urethral ureteroscopy laser lithotripsy (flexible cystoscope) on May 27. Postoperative analysis revealed that the stone was composed of xanthine.

**Fig. 1:**
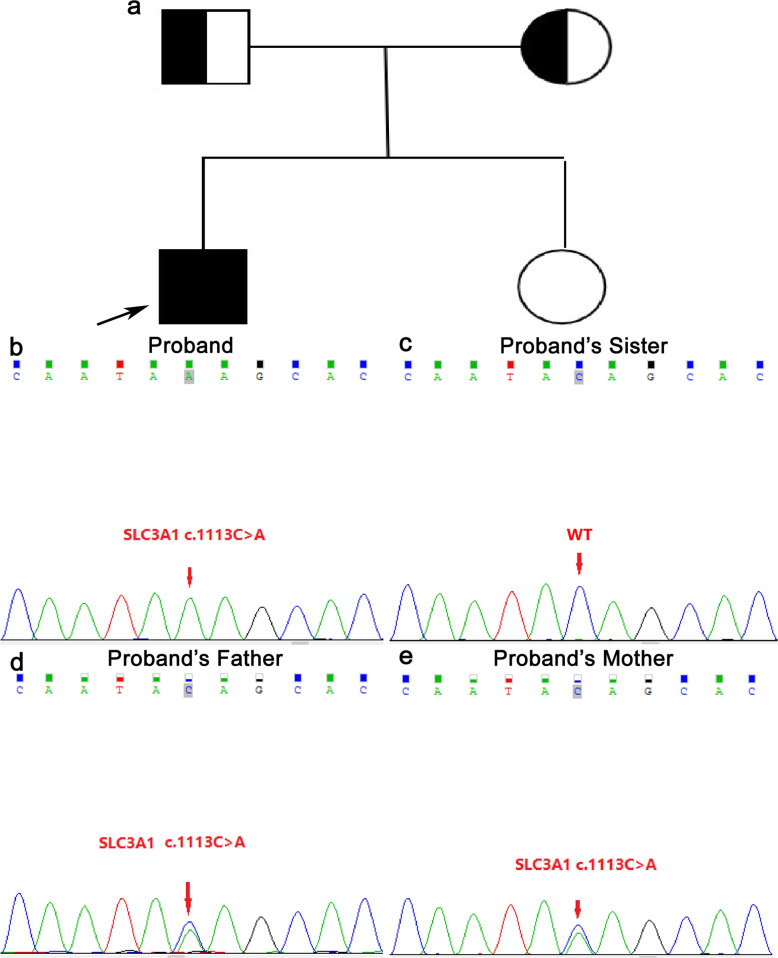
Genetic map of cystinuria and Sanger sequencing of the SLC3A1 mutation site. **a** Genetic map of cystinuria (the arrow indicates the proband; the parents and sister of the proband have no history of urinary calculi). **b** The c.1113C>A homozygous mutation in the SLC3A1 gene of the proband. **c** The SLC3A1 gene c.1113C>A of the proband’s sister (wild-type). **d** The c.1113C>A heterozygous mutation in the SLC3A1 gene of the proband’s father. **e** The c.1113C>A heterozygous mutation in the SLC3A1 gene of the proband’s mother

The patient’s blood metabolism test showed that his blood uric acid (661 $\upmu$mol/L) was significantly higher than the normal range (88–430 $\upmu$mol/L), which was inconsistent with the common symptoms of xanthine stones. Genetic testing by next-generation sequencing (NGS) carried out with the patient’s informed consent revealed a homozygous nonsense mutation (c.1113C> A, p.Tyr371*) in the *SLC3A1* gene. There are multiple reports of nonsense variants causing disease in the upstream and downstream of this variant [8, 9], thus implicating loss-of-function as one of the pathogenic mechanisms of the disease. Four cases of heterozygotes of this variant are reported in the gnomAD population database, while no homozygous individuals have been found. To clarify the source of the variant, Sanger sequencing of this site was performed on the patient, his parents and his sister. The patient’s parents were found to carry the heterozygous mutation (the parents were not in a consanguineous marriage), and the sister did not carry the mutation (Fig. 1). According to previous studies, pathogenic variants in the *SLC3A1* gene can cause cystinuria. The diagnosis of cystinuria was confirmed by performing 24-h urine metabolism analysis of cystine, which was found to be 644 mg/24 h (normal value <320 mg/24 h). After discharge from the hospital, the patient was treated with potassium hydrogen citrate sodium (2.5 g/time, 4 times/day), aluminum phosphate gel (20 g/time, 2 times/day), and cefdinir capsules (0.1 g/time, 3 times/day). Preventive measures including drinking sufficient amounts of water, exercising moderately, and restricted meat, fish, shrimp and other high-purine foods were ordered and reviewed regularly.

## Discussion

Composition analysis is the main method used to diagnose the nature of stones in patients with urinary calculi. In the case reported here, the stones removed from the patient were found to be composed of xanthine, although his blood uric acid content was elevated, which was not consistent with the common symptoms of xanthinuria. Therefore, we performed genetic analysis of a peripheral blood sample, which revealed a *SLC3A1* gene mutation known to be a pathogenic variant of cystinuria. This diagnosis was confirmed by the results of 24-h urine metabolism analysis. This is the first case report of cystinuria combined with xanthine stones.

Despite being an important symptom of cystinuria, not all patients develop cystine stones. A study of 39 members of a family showed that 15 members (average age 44 years) had elevated urine cystine excretion, while none of the family members reported symptoms of cystinuria or a history of stones. Only three of the family members were diagnosed with stone-forming cystinuria. Although the cause of stone-free cystinuria is not clear, the formation of cystine stones seems to be the result of polygenic factors. In addition to excessive cystine excretion in the urine, other factors may be involved in promoting or inhibiting the formation of stones [10]. It has also been reported that all patients with homozygous or compound/mixed heterozygous mutations of *SLC3A1* and *SLC7A9* have increased cystine excretion in urine, and 94% of these patients will go on to form stones [11]. The patient in this case had a homozygous mutation of the *SLC3A1* gene, and the urinary cystine content was found to be much higher than the normal value, but no cystine stones were formed, indicating that other factors inhibit the formation of cystine stones.

Most of the stones formed in patients with cystinuria are composed of cystine alone. However, up to 40% of patients will form mixed composition stones, including calcium oxalate, calcium phosphate or struvite [12]. Martins et al. found that adding cystine to undiluted human urine *in vitro* increased the growth rate of stone crystals by 52%, although electron microscope evaluation showed that the crystals were composed of calcium oxalate [13]. This finding revealed that cystine can promote the growth and aggregation of calcium oxalate crystals, just as the proposed mechanism of hyperuricosuria can promote the formation of calcium oxalate stones [14]. These results also indicate that cystinuria is a risk factor for non-cystine stones (mainly composed of calcium oxalate) [15].

Cystinuria is often accompanied by metabolic abnormalities [16]. Sakhaee et al. found that among patients with cystinuria, 18.5% had hypercalciuria, 22.2% had hyperuricuria, and 44.4% had hypocitraturia, possibly due to metabolic disorders of the kidneys [17]. It has also been reported that patients in the homozygous cystinuria group have a significantly higher incidence of hyperuricemia, but no hyperuricosuria. There are three possible reasons for this: uric acid-related pathways are activated and lead to hyperuricemia; the patient’s uric acid excretion is impaired, leading to hyperuricemia without hyperuricosuria; or high-purine diets may also cause hyperuricemia [18]. In the case reported here, the patient’s urinary cystine content was 644 mg/24 h, which is a typical cystinuria, although the stone was found to be compose of xanthine. These findings indicate that cystinuria may be a risk factor for the occurrence of xanthine stones. This may be related to the ability of cystine in the urine to promote xanthine stone formation. It may also be that cystine affects xanthine metabolism, causing activation of the related metabolic pathways. However, further investigations are required to fully clarify the cause of stones in cystinuria patients.

Genetic testing has become one of the main methods used to assist in the diagnosis of genetic diseases. Hereditary xanthinuria is predominantly caused by mutations in genes such as *XDH*, *MOCOS*, *MOCS1*, *MOCS2*, and *GPHN*. In these patients, xanthine metabolism is blocked, leading to the accumulation of excessive amounts of xanthine in serum and urine, and ultimately the formation of xanthine stones. In the case presented here, the stone was composed of xanthine. However, xanthinuria-related gene mutations were not detected in the genetic analysis, and this was ruled out as the cause of the xanthine stones. However, a homozygous nonsense mutation c.1113C> A (p.Tyr371*) in the *SLC3A1* gene was detected, which is the pathogenic gene that causes cystinuria [19, 20]. The diagnosis of cystinuria was confirmed by 24-h urine metabolism analysis. These findings indicate that homozygous mutations in this gene may affect the metabolism of xanthine.

In summary, genetic testing revealed a nonsense mutation in the *SLC3A1* gene in a patient with a stone composed of xanthine, which indicates the diagnosis of cystinuria. Our results not only demonstrate the potential of genetic testing as a basis for clinical diagnosis and treatment of cystinuria, but also provide a molecular basis for further research on the relationship between cystinuria and xanthinuria.

## Data Availability

All data generated or analysed during this study are included in this published article.
